# On the Side of the Commandments

**Published:** 1891-07-04

**Authors:** 


### The Hospital

A Weekly Journal of

Science, Medicine, Nursing, and Philanthropy.

*For Hospitals, Asylums, Infirmaries, Dispensaries, Medical Practitioners, Students, Nurses, and the Charitable Public.*

Vol. X.—No. 249.] [July 4, 1891.

### On the Side of the Commandments.

Lord Beaconsfield on a famous occasion said he preferred to be "on the side of the angels"; the President of the Sunday Society declared at Lord Brassey's last Saturday that he and his association were "on the side of the commandments." That is the very point at issue. The best and the most sensible people of this country have great respect for the commandments, and that for the best and most sensible of reasons. To many minds, both Christian and Jewish, the commandments of themselves constitute a noble ark of safety in a stormy and tempestuous time. It is true they impose certain uncomfortable checks upon a class of men, and even of women, who think nothing is quite so nice and pleasant as doing altogether as you please in all the affairs of life. But policemen and magistrates and prisons also act as uncomfortable checks upon people who are inclined to be too frisky in certain directions, though we never yet heard of any honest and sensible human being who objected to magistrates and policemen.

The Sunday Society does well to take the side of the commandments, and to tell the world its position. It is because many people have thought that the very reverse was the fact that they have opposed its activities. Our interest in the commandments as a journal must of necessity be more or less of the scientific kind. The particular commandment which the Sunday Society professes to support is of course the fourth, "Remember the Sabbath day to keep it holy. Six days shalt thou labour and do all that thou hast to do; but the seventh is the Sabbath of the Lord thy God; in it thou shalt do no manner of work." Thus the commandment runs. It was addressed in the first instance to the Jews. It is categorical in form, and prohibitory in intention. Nevertheless it contains within it a positive principle applicable and advantageous to all nations, at all times, and under all circumstances.

Physiology is a fruitful science. It teaches us that the energy of the human body, and the energy of the mind too, are like the ocean tides; they have their flow and their ebb. Let the strongest man in the world be taken as our typical example; he can work so many hours a day with advantage, and no more. The time comes when he must stop for rest and food; at longer intervals he must stop longer for more rest and sleep; and at still longer he must stop for still more extended periods of rest, and for a complete change of physical activities and mental conditions. Nature points out the proper breaks in daily work, for she makes men hungry. She also indicates the time for rest and sleep, for she enshrouds us in the mantle of night, and herself closes the curtains of our eyes in restful slumber. To our thinking also she confirms the religious ordinance which gives one day in seven for the complete dismissal of toil and complete change. It is as plain as possible to the unprejudiced physician that the devotion of every seventh day to absolute rest is markedly conducive to health. The man who works moderately every day and all day long does not need more than one day's rest in seven. But experience shows that one day in eight, one in nine, one in ten, or one in twelve or fourteen is not anything like so good for health as one in seven. The physiologist need not really concern himself to ask whether the Sabbath is of divine origin or not. What is of importance from his point of view is that one day in seven for rest answers all the purposes of work and health as well as, or better than, any other arrangement which has ever been tried on a large scale in any part of the world.

As a matter of fact it is pretty universally agreed, that one day in seven should be a day of freedom from toil. The question that really arouses controversy at the present time is not whether there shall be a Sabbath, but how the Sabbath shall be spent. Many persons spend it, by marked preference, entirely in religious exercises. They have been trained to that way of life from infancy; they love it; and they find their happiness in it. The ordered system of their life revolves round the Sabbath and all that it connotes as round a central sun. Will any wise and experienced man either sneer at such persons, or try "to show them a better way"? For them there is no better way. The purpose of their life is like that of King David, to "serve their generation according to the will of God," and then, like him, "to fall on sleep." Is there anything contrary to science, or to sound philosophy, in such a scheme of life? Most assuredly not! No man of science or mental philosopher, who has the least regard for his literary or judicial reputation will utter a single word of objection. If objection there be, it can only come from ignorance, inexperience, or folly.

But though vast numbers of people in this country heartily choose to spend their Sundays in the way here described, and though that way has our entire sympathy and practical support, we are convinced that it is neither wise, nor right, nor helpful to the cause of religion to strive to compel all persons thus to spend their Sunday. There is a certain note of Phariseeism in much of the Christianity of the present day. A manifest consciousness of superiority marks the devotees of all the churches and sects; of superiority as compared with one another, and of still greater superiority as compared with the non-religious world. It is the business of the physiologist to protest against this unscientific habit of mind; a habit as unchristian as it is unscientific. If science utters any one great truth it is this, that God is the God of the world and not merely of the churches; and that Jesus Christ, in whatever sense His Saviourship be accepted, is the Saviour of the world and not merely of the churches.

The outside world which the churches, with their shallow judgments, are so ready to call non-religious, is not really non-religious. Experience in all countries proves this contention. The great world knows God in a practical sort of way; knows Him, venerates Him, trusts Him, hopes for fuller light and a greater power of practical obedience. The physiologist claims liberty of conscience for this great outside world; freedom to serve God in its own way; to see Him in pictures and in sculpture; to admire Him in the forest trees and in the flight and song of birds; to feel Him in the soothing calm of the summer air, and in the myriad gentle sounds of the meadows, the cornfields, and the running brooks.

The essential factor in the Sabbath is not that it shall be a day of stated public religious services, but that it shall be a day of bodily and mental rest, of new creation, of innocent pleasure, of sober self-recollection: a day in which a quiet thinker shall forget that a large part of his life is necessarily devoted to the toiling for bread, and shall remember that "man does not live by bread alone, but by every word that proceedeth out of the mouth of God": every word of moral wisdom; but also every word of scientific truth, of beauty in art, of social good behaviour, of personal joyfulness in abundance of life and all that appertains thereunto.

Is it not passing strange that the physiologist should have to defend religion against so many of its professors? Should have to contend for the authority of God over all the world against those religious persons who strive to limit His authority to a part of the world? Should have to claim for every child of man his inheritance in the divine love, the divine guidance, and the divine protection; and his right to move about without restriction in the freedom of the Divine household? Narrow sabbatarians are usually very self-satisfied and very self-righteous persons. If there is anything which the New Testament hates it is self-righteousness; and the Old Testament, if possible, hates it still more. The Sunday Society, in striving to throw open every place and every opportunity of innocent recreation and pleasure to the whole population, is not only serving the causes of bodily and mental health, and of good government, but it is serving the cause of reasonable religion in the most decisive way. If any ecclesiastical religionist is in doubt on this point let him go back to the first principles of his faith and find reason and intelligent conviction there.

